# Impact of Pre-Mortem Factors on Meat Quality: An Update

**DOI:** 10.3390/foods10112749

**Published:** 2021-11-10

**Authors:** Gen Kaneko

**Affiliations:** School of Arts & Sciences, University of Houston-Victoria, Victoria, TX 77901, USA; kanekog@uhv.edu

Meat quality is closely associated with the chemical composition of skeletal muscle and is therefore influenced by the pre-mortem metabolic state of skeletal muscle tissue. Muscle metabolism is affected by various pre-mortem factors such as diet, age, genetic background, and environmental temperature. The importance of muscle metabolism has been increasingly recognized as an intermediate element that links meat quality and pre-mortem factors (i.e., growth conditions) in meat science [1,2,3].

This special issue of Foods, “Impact of Pre-Mortem Factors on Meat Quality” (ISSN 2304-8158), aims to compile the recent literature with a focus on pre-mortem factors, muscle metabolism, and meat quality. It includes nine research articles about various types of meat (beef [4], lamb [5], pork [6], chicken [7,8,9], goat [10,11] and fish [12]) as well as one review article about beef quality [13]. These articles, while their aims are different, provide an accurate representation of the current frontier of meat science and the direction in which it is heading. This editorial article is written to introduce three aspects of food science, highlighted by articles in this special issue.

The first keyword is sustainability. A sustainable food system is a system that “delivers food security and nutrition for all in such a way that the economic, social and environmental bases to generate food security and nutrition for future generations are not compromised” [14]. The practical concept of sustainability in the food industry includes the maximum utilization of known materials, the identification of new alternative foods, ensuring economic stability for producers, and food safety/security. In this regard, most food science studies should be of sustainable value, but studies on indigenous chickens [7], algae or insect supplementation in chicken diets [8], and milk replacers [10,11] clearly offer sustainable solutions to current problems in meat science. The continued characterization of novel pre-mortem factors that influence meat quality contributes to optimizing the growth conditions of animals.

The second aspect is the emergence of the omics approach, which has allowed us to understand the overall changes in muscle metabolism induced by pre-mortem factors. Metabolomics is probably the most common omics technology in meat science because meat samples are often not “fresh” enough to be analyzed by proteomics or transcriptomics. In their novel and highly relevant study, Biondi et al. (2019) analyzed the effect of diet on a microbiome using lamb meat [5]. Tuell et al. (2020) demonstrated that metabolomics is sensitive enough to predict the effect of photoperiod, which seems indirect compared to other factors such as diets and temperature, on oxidative stability in broiler fillet [9]. The omics technologies are not yet applicable on-site, mainly due to their high cost but will be introduced to assess meat quality [15].

The last aspect is the application of advanced computational procedures. In particular, the accumulation of data from omics studies has facilitated the use of computational procedures in meat science, and in fact, some articles in this special issue are closely related to the omics “big data” approaches from the same group [16,17,18]. Research articles in this special issue analyze factors that affect meat quality via mathematical modeling [4], machine learning [12], and correlation-clustering analysis [6]. One review article summarizes the effect of diet and genetics on beef quality characteristics [13]. Along with the prevalence of omics approaches, greater computational efforts will be required in meat science.

Due to the inclusion of rigorous and well-researched articles, this special issue offers a valuable contribution to meat science, highlighting the potential role of omics and computational analysis to further advance meat science in a sustainable manner. A possible addition to current research efforts would be the integrated experimental design and writing style, which facilitate the use of the systematic approach. Systematic reviews have become an important method to provide evidence-based interventions in medicine, but their application has been, and is still, uncommon in meat science. This is partly because of the diverse nature of muscle metabolism—results acquired from a specific species cannot be directly compared with those from other species. However, that is indeed the reason why data from a single study should be effectively utilized. A concerted effort is necessary to move the field forward, providing promising protein sources for humans in the present era of food security concerns.

## Data Availability

Not applicable.
